# Assessment of Bone Age Based on Hand Radiographs Using Regression-Based Multi-Modal Deep Learning

**DOI:** 10.3390/life14060774

**Published:** 2024-06-18

**Authors:** Jeoung Kun Kim, Donghwi Park, Min Cheol Chang

**Affiliations:** 1 Department of Business Administration, School of Business, Yeungnam University, Gyeongsan-si 38541, Republic of Korea; kimjk70@yu.ac.kr; 2 Seoul Spine Rehabilitation Clinic, Ulsan-si, Republic of Korea; bdome@hanmail.net; 3 Department of Rehabilitation Medicine, College of Medicine, Yeungnam University, Daegu 42415, Republic of Korea

**Keywords:** bone age, deep learning, regression, radiograph, clinical data

## Abstract

(1) Objective: In this study, a regression-based multi-modal deep learning model was developed for use in bone age assessment (BAA) utilizing hand radiographic images and clinical data, including patient gender and chronological age, as input data. (2) Methods: A dataset of hand radiographic images from 2974 pediatric patients was used to develop a regression-based multi-modal BAA model. This model integrates hand radiographs using EfficientNetV2S convolutional neural networks (CNNs) and clinical data (gender and chronological age) processed by a simple deep neural network (DNN). This approach enhances the model’s robustness and diagnostic precision, addressing challenges related to imbalanced data distribution and limited sample sizes. (3) Results: The model exhibited good performance on BAA, with an overall mean absolute error (MAE) of 0.410, root mean square error (RMSE) of 0.637, and accuracy of 91.1%. Subgroup analysis revealed higher accuracy in females ≤ 11 years (MAE: 0.267, RMSE: 0.453, accuracy: 95.0%) and >11 years (MAE: 0.402, RMSE: 0.634, accuracy 92.4%) compared to males ≤ 13 years (MAE: 0.665, RMSE: 0.912, accuracy: 79.7%) and >13 years (MAE: 0.647, RMSE: 1.302, accuracy: 84.6%). (4) Conclusion: This model showed a generally good performance on BAA, showing a better performance in female pediatrics compared to male pediatrics and an especially robust performance in female pediatrics ≤ 11 years.

## 1. Introduction

The assessment of bone age is a clinical method used to determine the stage of skeletal maturation in children [1]. Throughout an individual’s lifetime, bones undergo significant changes in shape, with the most notable changes occurring during the growth period at an early age [2]. In humans, hands are composed of 27 bones, including carpal, metacarpal, and phalange bones [3]. As a result, the hand is an ideal part of the body for bone age assessment by radiographic imaging due to its high number of bones in a relatively small space requiring low levels of radiation [3].

In clinical practice, a bone age assessment (BAA) is typically carried out by comparing radiographs of the non-dominant hand with a reference atlas containing known sample bones [4]. The most well-known atlases for measuring bone age are the atlas of Greulich and Pyle and that of Tanner–Whitehouse [5]. Greulich and Pyle developed an atlas based on radiographs of hand regions that exhibited the most distinctive chronological changes throughout the aging process [5]. Subsequently, Tanner and Whitehouse created a more extensive atlas of hand radiographs characterizing age-wise morphological changes in bones [5]. However, the methods based on these atlases are time-consuming and depend on the expertise and experience of physicians, making them susceptible to observer variability.

Deep learning is an advanced machine learning approach that involves the use of a large number of hidden layers to build artificial neural networks with structures and functions similar to those of the human brain [6,7,8]. It can learn from unstructured and perceptual image data. Deep learning is currently widely utilized as an analytical method in a variety of fields, from research and business to the arts, demonstrating excellent performance in image analysis [9]. In particular, in the field of medicine or healthcare, deep learning is extensively being applied to identify features in image data from which to make diagnoses, as well as to predict disease prognosis [6,7,8]. Owing to the characteristics of deep learning, it has been applied to BAA, and its usefulness has previously been demonstrated [10,11,12,13].

Recent advancements in CNNs have led to significant improvements in image tasks and focus on enhancing model performance by effectively combining features from different layers or modalities, including transformer-based models [14], efficient architecture [15,16], multi-scale feature fusion [17] and early and late fusion [18]. Feature fusion in CNNs enhances performance by aggregating information from diverse sources, such as multi-modal data (e.g., RGB and depth) or different network layers [16]. This allows for a more comprehensive representation of input data, capturing both fine-grained details and the global context, leading to improved performance in various tasks [19]. For example, multi-scale feature fusion methods like the Swin Transformer [20] have demonstrated significant improvements in object detection and semantic segmentation. This adaptability leads to better performance compared to using single feature types, further highlighting the effectiveness of feature fusion in modern CNN architectures.

Previous research on deep learning-based BAA has primarily used the following two approaches: classification models and regression models [10,11,12,13,21]. Studies by Lee et al. (2017) [12] and Lee et al. (2021) [13] highlight the effectiveness of classification-based approaches, yet the opaqueness of these models can limit their interpretability, potentially preventing clinicians from comprehending the rationale behind diagnostic results. Regression-based BAA models employ deep learning to predict a continuous variable, such as bone age, from hand radiographic images [10,21]. Regression models offer a more clinically relevant approach to assessing bone maturity by outputting a continuous numerical age estimate rather than categorizing images into discrete classes like classification models [10,21]. While both types of models are susceptible to class imbalance, deep-learning regression models are relatively better suited for handling imbalanced data. By predicting specific values instead of assigning inputs to distinct class labels, regression models can mitigate biases towards frequently represented classes and maintain more consistent performance across imbalanced data distributions.

In addition, many of the previously developed BAA models did not use real clinical data, hindering the accurate assessment of bone age [10,12,21]. In this respect, the multi-modal deep learning model is expected to increase the accuracy of BAA in the deep learning model.

In the present study, a regression-based multi-modal deep learning model that utilizes hand radiographic images, patient gender, and chronological age as input features was developed with the aim of improving the accuracy of BAA. The diagnostic performance of the proposed model was validated to ensure its reliability and robustness in clinical settings.

## 2. Materials and Methods

### 2.1. Subjects

The study protocol was approved by the institutional review board of Ulsan University Hospital, which waived the requirement for written informed consent owing to the retrospective nature of this study. This study was conducted in accordance with the Declaration of Helsinki.

We collected images from 2974 pediatric patients who underwent left-hand radiography at Ulsan University Hospital between March 2010 and August 2023 and distributed the number of patients evenly by age. Table 1 shows the number of patients by age.

Patients younger than two years of age were excluded from the study because the program that was used in this study was developed according to the Greulich–Pyle (GP) method [5], which is not suitable for the evaluation of bone age in pediatrics younger than one year of age. We also excluded pediatric patients with a history of bone fracture or surgery in their left hand, as well as patients with confirmed genetic abnormalities, such as Downs syndrome or Klinefelter syndrome. The mean (±SD) age of the patients was 9.4 ± 2.5 years (range, 1–17 years), comprised 812 male and 2162 female patients.

A radiologist with over 20 years of clinical experience determined the bone age based on the left-hand radiograph of each included patient using the GP method.

### 2.2. Deep Learning Algorithm

Python 3.8.10, SciKit-Learn 0.24.2, and TensorFlow 2.10.1 with Keras were used to develop the deep learning model for BAA.

To address the challenges posed by the imbalanced distribution of gender and age within the training dataset and the relatively limited number of images available, a multi-modal deep learning model was implemented. The model utilized both hand radiographic images and essential clinical data of pediatrics, including gender and date of birth, in the training process. This approach aims to enhance the model’s learning efficiency and diagnostic accuracy by integrating diverse data modalities.

Leveraging a multi-modal approach, we significantly improved the model’s performance by simultaneously processing hand radiographic images through the EfficientNetV2S CNN model and clinical data via a simple DNN model. The model’s strength lies in the strategic integration of outcomes from both sources in the final training stage, harnessing the distinct advantages of image and clinical data. This synergy enhances the model’s robustness, demonstrating the efficacy of multi-modal frameworks in combining diverse data types for better diagnostic precision.

While MAE and RMSE are also commonly used loss functions for regression models, the MSE (mean squared error) was chosen for this study. The MSE is calculated as follows:

MSE = (1/n) × Σ(y_i_ − ŷ_i_)^2^

where

n: the total number of data points;
y_i_: the actual (true) value for the i-th data point;
ŷ_i_: the predicted value for the i-th data point;
Σ: the summation symbol, indicating that we summed over all data points.

**Figure 1:**
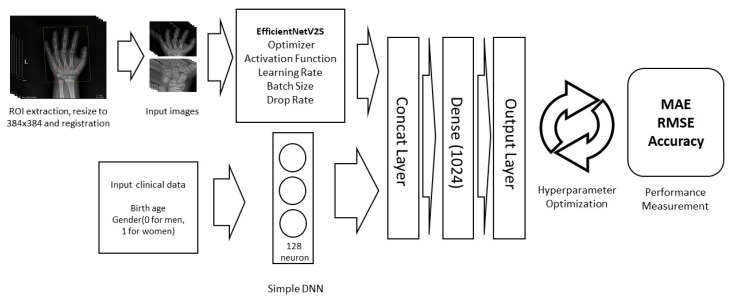
Schematic representation of the process used for assessing bone age (ROI: region of interest; DNN: deep neural network; MAE, mean absolute error; and RMSE, root mean squared error).

The squaring of the errors in the MSE formula makes it sensitive to outliers, which is particularly relevant in the context of bone age assessment. Large deviations in bone age diagnoses can have significant clinical implications, potentially leading to inappropriate treatment decisions. Figure 1 shows the training details of our bone age diagnosis model.

### 2.3. Statistical Analysis

Statistical analyses were performed using Python 3.8.10 and Scikit-Learn version 0.24.2. The performance of the bone age diagnosis model, which employs the deep learning regression model, was evaluated through MAE and RMSE. Additionally, to assess clinical accuracy, we compared the model-predicted bone ages with specialist diagnoses. The agreement between the model’s predicted bone age and the specialist’s assessment was defined as a difference of ≤1 year, which was considered accurate [12]. Conversely, a difference of >1 year between the model and the specialist was considered a disagreement, indicating inaccuracy in the model’s prediction.

## 3. Results

The proposed model exhibited robust performance on validation data, with an overall MAE of 0.410, RMSE of 0.637, and accuracy of 91.1%. Subgroup analysis revealed higher accuracy in females ≤11 years (MAE: 0.267, RMSE: 0.453, accuracy: 95.0%) and >11 years (MAE: 0.402, RMSE: 0.634, accuracy: 92.4%), compared to males ≤13 years (MAE: 0.665, RMSE: 0.912, accuracy: 79.7%) and >13 years (MAE: 0.647, RMSE: 1.302, accuracy: 84.6%). According to these results, the model demonstrated a good performance in female pediatric patients, with an especially high performance in female pediatrics of ≤11 years. Details of the sample size and the developed model are provided in Table 2 and Table 3.

## 4. Discussion

The multi-modal model for use in BAA developed in this study achieved an overall accuracy of 91.1% on a validation set of 597 images. The model showed excellent performance for females aged 11 years or younger (accuracy: 95.0%, MAE: 0.267, RMSE: 0.453) and maintained a robust performance for females of >11 years (accuracy: 92.4%, MAE: 0.402, RMSE: 0.634). However, compared to females, the model’s performance was relatively low for males ≤ 13 years (accuracy: 79.7%, MAE: 0.665, RMSE: 0.912) and >13 years (accuracy: 84.6%, MAE: 0.647, RMSE: 1.302).

The discrepancies in the model performance between the female and male subgroups can be largely attributed to the limited sample size of male pediatrics (812 images) compared to females (2162 images), with an even smaller subset of males older than 13 years (126 images). This imbalance may lead to an underrepresentation of the variability in male skeletal maturation patterns, making it challenging for the model to learn and generalize effectively for this demographic group [19]. In contrast, the larger female sample size allowed the model to better capture the subtleties and variations in female bone development, resulting in higher accuracy and lower error rates. The limited male sample size may also increase the model’s sensitivity to outliers or noise, contributing to higher MAE and RMSE values.

To date, a number of studies have used deep learning to predict bone age in hand radiographs [10,11,12,13,19,20]. In 2017, Spampinato et al. used deep learning methods to estimate bone age in hand radiographs [20]. As a result of evaluating and analyzing several algorithms on public data, they found that the average error in bone age was 9.6 months, suggesting a direction for bone age evaluation using deep learning. In 2017, Lee et al. developed a fully automated bone age assessment system based on transfer learning using convolutional neural networks that initially segmented the palm region of the hand radiographs from the background using a convolutional neural network and then utilized the entire map as the input [12]. The RMSE of their developed system was 0.93 years for females and 0.82 years for males. In 2017, Kim et al. used deep learning methods to analyze 18,940 left-hand radiographs evaluated by the GP method in Korean children [11]. In that study, the automated software system demonstrated a concordance rate of 69.5% and a significant correlation with baseline bone age (r = 0.992; *p* < 0.001). In 2020, Pan et al. used GP-independent deep learning for the automated assessment of bone age by training an algorithm to estimate chronological age using bone morphology on a training set of over 10,000 pediatric trauma hand radiographs [19]. The MAE of the resulting model was 12.9 months.

The multi-modal model developed in the present study demonstrated a remarkable performance despite being trained and validated on a relatively small dataset of 2974 images. The model achieved an impressive overall accuracy of 91.1%, highlighting its efficiency in learning and generalizing from a compact dataset. Moreover, the model exhibited exceptionally low error rates for female pediatric patients, with an MAE of 0.267 and RMSE of 0.453 for females aged 11 years or younger and an MAE of 0.521 and RMSE of 0.608 for females of >11 years. The error rates in this study were substantially lower than those reported by Lee et al. (2017) and Pan et al. (2020) despite their use of considerably larger datasets consisting of (8325 and 15,129 subjects, respectively) [12,19]. The multi-modal approach applied to develop our model, incorporating both imaging data and clinical information, likely contributed to the model’s success in learning from a limited number of samples by leveraging complementary information from different sources. This model’s relatively robust performance across age groups, despite the smaller dataset, further emphasizes its adaptability and generalizability for real-world clinical applications. In summary, the proposed multi-modal BAA model’s high performance, achieved with a relatively small dataset, highlights its effectiveness and potential for practical implementation in clinical settings, particularly when large-scale datasets may not be readily available.

Although the proposed BAA model demonstrated a good performance overall, the accuracy of bone age assessment for males was relatively lower compared to that of females. This discrepancy can be attributed to the imbalance in the training and validation data, with a significantly smaller number of male samples available. To address this issue and further enhance the model’s performance, future research should focus on acquiring additional data, particularly for male pediatrics. By expanding the dataset and ensuring a more balanced representation of both genders, it is expected that the proposed model’s accuracy and generalizability will improve, enabling more reliable and consistent bone age assessment across all pediatrics.

Additionally, the proposed bone age diagnosis model is regression-based, which inherently prevents the direct calculation of sensitivity, specificity, and AUC. This limitation restricts the comprehensive validation of the model’s performance due to its inability to utilize various performance metrics. Another limitation of our study is the imbalance in the training data. The number of female patients in our dataset is nearly three times higher than that of male patients. We attempted to mitigate this data bias by incorporating patient information (birthdate and gender) alongside hand X-ray images during training, aiming to improve diagnostic accuracy. However, we were not able to fully eliminate this bias. Future research should focus on acquiring a more balanced dataset to address this limitation. We acknowledge this as a potential area for improvement and will explore alternative evaluation strategies in future work.

## Figures and Tables

**Table 1:** Number of images used for model training and validation.

**Age**	**1**	**2**	**3**	**4**	**5**	**6**	**7**	**8**	**9**	**10**	**11**	**12**	**13**	**14**	**15**	**16**	**17**	**Total**
**Male**	1	0	8	17	43	26	70	73	67	93	102	117	69	58	35	27	6	812
**Female**	8	7	13	22	40	119	249	352	396	443	250	173	52	25	9	4	0	2162
**Total**	9	7	21	39	83	145	319	425	463	536	352	290	121	83	44	31	6	2974

**Table 2:** Bone age diagnosis model performance.

Sample size	2385 images (79.9%) for training; 597 images (20.1%) for validation; and a total of 2974
Sample size by gender and age	- Female (≤11 years) training 1519, validation 380, total 1899 images - Female (>11 years) training 210, validation 53, total 263 images - Male (≤13 years) training 548, validation 138, total 686 images - Male (>13 years) training 100, validation 26, total 126 images
Model details	- Multi-modal model with EfficientNetV2S CNN for images and DNN for clinical data - Optimizers: RMSProp and Adam - Activation function: ReLU - Learning rate 1 × 10^−5^, batch size 8 - Regularization: Batch Normalization and dropout - ROI image resized to (384 × 384) - Clinical data as input: birth date and gender
Model performance (validation data)	- Overall: MAE 0.410, RMSE 0.637, accuracy 91.1% - Female (≤11 years, 380 images): MAE 0.267, RMSE 0.453, accuracy 95.0% - Female (>11 years, 53 images): MAE 0.402, RMSE 0.634, accuracy 92.4% - Male (≤13 years, 138 images): MAE 0.665, RMSE 0.912, accuracy 79.7% - Male (>13 years, 26 images): MAE 0.647, RMSE 1.302, accuracy 84.6%

CNN, convolutional neural network; RMSProp, root mean squared propagation; Adam, adaptive moment estimation; ReLU, rectified linear units; ROI, region of interest; MAE, mean absolute error; and RMSE, root mean squared error.

**Table 3:** Layer types and parameters in bone age diagnosis model.

Layer (Type)	Output Shape	Number of Parameter
Input_cl (input layer)	[(None, 1)]	0
Input_img (input layer)	[(None, 384, 384, 3)]	0
Dense	(None, 128)	256
Efficientnetv2 s (functional)	(None, None, None, 1280)	20,331,360
Batch_normalization	(None, 128)	512
GlobalAveragePooling2D	(None, 1280)	0
Dense	(None, 256)	33,024
Concatenate	(None, 1536)	0
Dropout	(None, 1536)	0
dense_2 (dense)	(None, 1024)	1,573,888
dense_3 (dense)	(None, 1)	1025
Total params: 21,940,065 (83.69 MB)		
Trainable params: 21,785,937 (83.11 MB)		
Non-trainable params: 154,128 (602.06 KB)		

## Data Availability

Some or all data, models, or codes generated or used during this study are available from the corresponding author on request.
